# A Letter from Philadelphia

**Published:** 1860-02

**Authors:** 


### Correspondence.

Messrs. Editors:—I have just come across an interesting notice by a Paris correspondent of a New York paper, of the new French discovery or method of producing anæsthesia, and from which I make a few extracts, presuming they may interest your readers and induce experiments.

The writer is hopeful that in this method a welcome rival to chloroform has been found, and one that is free from all risks; and suggests that it may be the key by which animal magnetism and kindred mysteries can be explained.

"Take any small brightish object, a knife blade, penholder, half dime, for example: Hold it before a person's eyes, a little within and above the range of his or (which is better) her easy vision, so that when she looks at it, and thinks of it fixedly, as she must be engaged to do, she will have to squint convergently, upwardly and abominably, and the chances are, that in a space of from one to three minutes, she will have passed into a state of unnatural sleep, nervous sleep, artificial catalepsy—what two weeks ago we would have styled (with more or less of a sneering tone, most of us), magnetic sleep, what we now solemnly style *Hypnotism.* Now you may raise her two feet from the floor, tickle the soles of them, pull out the eyebrows, set out one arm like a pump-handle, throw up the other like a flagstaff, cut off one of them if need be, and she will not move, complain, or feel fatigue or pain. When you have experimented sufficiently, blow upon her eyes, rubbing the latter gently if you choose, with your fingers, and she will wake up utterly unconscious of all that has happened.

"All medical Paris is busy now with experiments in Hypnotism. * * * * * * * * Dr. Broca, an eminent surgeon here, at the suggestion of Dr. Azram of Bordeaux, who had been set to look into the matter by reading a book of Dr. Braid, of Manchester, England, published in 1843—Dr. Broca, I say, began his experiments last month, in one of them performing what would otherwise have been a painful operation on a patient who was utterly unconscious of it till it was over, and she restored to her natural state. He made a report of his experiments, which Velpeau, one of the greatest and most incredulous, tough old surgeons of the world, consented to read before the Academy of the Sciences. Velpeau is too careful of his great reputation, as well as too careful and cautious a man to countersign Dr. Broca's note. But the mere fact of his reading it immediately gave its statements a certain authority. It was as if he had said to all the members of the profession: 'Here, these experiments are respectable; you can go on and make more without risking your little professional dignities and reputations, and run of practice. I, Velpeau, have said it.'

" And now all hands, Trousseau, Nelaton, and all the lesser and little ones are setting their private patients and inmates of the hospitals asquinting inwardly, upwardly and abominably to a cataleptic degree, and under the new title (invented by Braid in 1843), of *hypnotism* (which is Greek *vrvos*, for sleepism, nothing more), are seriously investigating facts."

Who says we are not an enterprising—an *expansive* people, and that *our profession* is not a power in the land?

Look for a moment at the extent of its representation abroad. Nearly all the crowned heads of Europe depend upon American dentists for the care of their teeth, and almost every capitol of Europe has its representative of American dentistry. This surely is something to be proud of professionally; but the word is still "ONWARD," for, as I have just learned, the dominions of the "brother of the sun and moon" is to be honored with the presence of an American dentist.

A gentleman attached to the American embassy in China, has written home to a friend in this city, asking him to prevail upon some good dentist to go out there, as the European and American residents—of whom it seems there is quite a large number—greatly need and desire his services and guarantee ample remuneration; and per consequence, a young practitioner of this city is now, I am informed, preparing to go.

We may now, I think, with much reason, soon expect a similar call from Japan.

A good many inquiries are made in regard to the nature of the settlement between the Hard Rubber Company and E. A. L. Roberts, on the subject of the patent for vulcanized rubber for dental purposes.

Many questions are asked in reference to the validity of the patent, and whether it has been established in a court of justice, etc. Can you or any of your readers answer?

Yours, O. U. C.

PHILA., Jan. 12, 1860.

